# Formulation of Honokiol- and Magnolol-Loaded Nanoemulsions for Head and Neck Cancer Adjuvant Therapy: Evaluation of Radiation Sterilization Effects on Active Substance Properties

**DOI:** 10.3390/ijms26168032

**Published:** 2025-08-20

**Authors:** Katarzyna Dominiak, Aleksandra Gostyńska-Stawna, Agnieszka Sobczak, Jarosław Paluszczak, Aneta Woźniak-Braszak, Mikołaj Baranowski, Paweł Bilski, Barbara Wicher, Ewa Tykarska, Anna Jelińska, Maciej Stawny

**Affiliations:** 1 Chair and Department of Pharmaceutical Chemistry, Poznan University of Medical Sciences, Rokietnicka 3, 60-806 Poznan, Poland; katarzyna.dominiak@student.ump.edu.pl (K.D.); asobczak@ump.edu.pl (A.S.); ajelinsk@ump.edu.pl (A.J.); mstawny@ump.edu.pl (M.S.); 2 Chair and Department of Pharmaceutical Biochemistry, Poznan University of Medical Sciences, Rokietnicka 3, 60-802 Poznan, Poland; paluszcz@ump.edu.pl; 3 Functional Materials Physics Division, Faculty of Physics, Adam Mickiewicz University, Uniwersytetu Poznańskiego 2, 61-614 Poznan, Poland; aneta.wozniak-braszak@amu.edu.pl (A.W.-B.); mikolaj.baranowski@amu.edu.pl (M.B.); 4 Department of Experimental Physics of Condensed Phase, Faculty of Physics and Astronomy, Adam Mickiewicz University, Uniwersytetu Poznańskiego 2, 61-614 Poznan, Poland; pawel.bilski@amu.edu.pl; 5 Department of Chemical Technology of Drugs, Poznan University of Medical Sciences, Rokietnicka 3, 60-802 Poznan, Poland; bwicher@ump.edu.pl (B.W.); etykarsk@ump.edu.pl (E.T.)

**Keywords:** radiation sterilization, intravenous lipid emulsion, NMR, EPR, honokiol, magnolol

## Abstract

Honokiol (HON) and magnolol (MAG), structural isomers from *Magnolia officinalis*, exhibit notable anticancer activity, particularly against head and neck squamous cell carcinoma (HNSCC). However, due to their high lipophilicity, their intravenous administration is challenging. This study aimed to develop HON- and MAG-loaded intravenous (IV) nanoemulsions using commercial lipid preparations with varying fatty acid compositions. The formulations were physicochemically characterized and evaluated in vitro using FaDu and SCC-040 HNSCC cell lines. HON and MAG were sterilized via ionizing radiation at doses of 25, 100, and 400 kGy. Their suitability for IV use was assessed through PXRD, DSC, TGA, EPR, FT-IR, NMR, and HPLC analyses. All formulations met safety criteria for IV administration, with mean droplet diameters below 241 nm and encapsulation efficiencies exceeding 95%. They significantly reduced cancer cell viability, with a synergistic effect observed in combined HON and MAG formulations compared to single-compound nanoemulsions. Clinoleic-based formulations showed enhanced anticancer efficacy, likely due to the pro-apoptotic properties of oleic acid. Notably, radiation sterilization at the standard 25 kGy dose preserved the thermal, crystalline, and structural stability of HON and MAG, whereas higher doses (400 kGy) induced degradation. Although free radicals were detected via EPR, their transient nature and rapid decay confirmed the method’s safety. HON/MAG-loaded nanoemulsions exhibited strong anticancer potential, while radiation sterilization at 25 kGy ensured sterility without compromising stability. These findings provide a preliminary in vitro basis for future in vivo studies investigating HON and MAG as potential adjuvant therapies for HNSCC.

## 1. Introduction

Honokiol (HON) and magnolol (MAG) are structural isomers isolated from various parts of *Magnolia officinalis* including the bark, seed cones, and leaves [1]. These compounds are known for their strong antioxidant, anti-inflammatory, and anticancer properties, making them promising candidates for therapeutic use in various clinical applications [2,3]. Numerous studies have explored HON’s effects on various cancers, including breast, ovarian, lung, liver, and glioblastoma, as well as individual studies on colon cancer, tongue cancer, nasopharyngeal carcinoma, bone cancer, and melanoma, demonstrating its broad anticancer potential [4]. MAG has been less extensively studied, though its potential in breast cancer treatment has been investigated by Elhabak et al. [5] and Wang et al. [6].

Our recent study confirmed the anticancer activity of HON and MAG in head and neck squamous cell carcinoma (HNSCC). Both compounds reduced the viability of SCC-040, wild-type FaDu, and even FaDu cisplatin persister cells by regulating the cell cycle and apoptosis. These findings highlight their potential as promising candidates for head and neck cancer therapy, particularly as adjuvant agents in overcoming chemoresistance [7]. Developing a safe and effective dosage form is essential to fully realize and accurately assess the therapeutic potential of these agents in vivo. Over the past decade, nanotechnology has significantly advanced drug delivery, particularly in the formulation of intravenous (IV) nanoemulsions designed to enhance the bioavailability and efficacy of lipophilic compounds [8]. Nanoemulsions offer a promising drug delivery approach, especially for lipophilic plant-derived compounds like HON and MAG, which have poor water solubility [9]. Incorporating these compounds into a stable and biocompatible carrier system, such as Clinoleic or Lipidem—commercial IV nanoemulsions composed of soybean oil and olive oil or soybean oil, medium-chain triglycerides, and omega-3 fatty acid triglycerides—presents an interesting strategy. Using commercial nanoemulsions as lipid carriers enables precise dose control of active ingredients and ensures bioavailability, bypassing the first-pass effect. However, IV administration requires a sterile product, making the sterilization process a critical step that must be carefully optimized to ensure safety without compromising the stability or efficacy of the active compounds. Since commercial IV nanoemulsions are sterile, identifying an effective sterilization method for the active compounds before their incorporation would enable the development of a viable IV formulation. Among available sterilization methods, radiation sterilization is suitable for active substances in solid form [10].

This study aimed to confirm the antitumor potential of commercial intravenous nanoemulsions containing HON, MAG, and their combination against HNSCC. Additionally, a radiation sterilization method for the active compounds was proposed, and the sterilized active substances were thoroughly assessed to evaluate their suitability for the preparation of sterile IV formulations for future in vivo studies.

## 2. Results and Discussion

### 2.1. Formulation and Characterization of Nanoemulsions

Based on our previous study, HON and MAG have demonstrated significant potential as adjuvant therapy candidates for treating head and neck cancer [7]. However, further research is needed to develop an appropriate drug formulation that would enable the effective delivery of these compounds to the target site. In this study, we incorporated HON and MAG, as well as their combination (HON-MAG), into commercial lipid emulsions using a previously developed low-energy horizontal shaking process with some modifications [11].

Two commercial nanoemulsions, Clinoleic and Lipidem, were selected for this study. Clinoleic consists of a mixture of 20% soybean oil and 80% olive oil, while Lipidem contains 40% soybean oil, 50% medium-chain triglycerides, and 10% omega-3 fatty acid triglycerides. This selection was based on the need to develop formulations with varying omega-3, -6, and -9 fatty acid contents.

Candiloro et al. [12] reported that head and neck cancer patients treated with olive-oil-based parenteral nutrition (containing Clinoleic as a lipid source) exhibited enhanced function of both the innate (natural killer cells and monocytes) and adaptive (both CD4 and CD8 cells) arms of the immune response. According to the authors, this may favor an antitumor response compared to patients receiving parenteral nutrition based on 43% soybean oil, 35% olive oil, and 22% fish oil enriched with omega-3 fatty acids.

Omega-6 and omega-9 fatty acids tend to have proinflammatory effects, and as shown by Candiloro et al., this proinflammatory activity can be beneficial in enhancing antitumor immune responses in vivo [12]. On the other hand, unlike omega-6 fatty acids (found in soybean oil) and omega-9 fatty acids (found in olive oil), which are associated with the activation of different inflammatory pathways, omega-3 fatty acids primarily attenuate inflammatory responses. These effects are mediated through the modulation of molecular pathways involved in inflammation, mainly driven by specialized pro-resolving mediators, which are bioactive metabolites derived from omega-3 fatty acids [13]. Omega-3 fatty acids have demonstrated protective properties in animal models of inflammatory diseases [14,15]. They are also recognized as pharmaconutrients that enhance the effectiveness of anticancer therapy and contribute to improved overall survival in cancer patients [16]. Moreover, in head and neck cancer patients requiring clinical nutrition, immunonutrition enriched in omega-3 fatty acids significantly reduced both overall postoperative complications and infectious complications [17]. Finally, recent studies have shown that fatty acids such as docosahexaenoic acid (omega-3) and linoleic acid (omega-6) may influence the response of cancer cells to chemotherapy by affecting the levels of microRNAs known as tumor suppressors [18].

These findings led us to formulate nanoemulsions based on two types of commercial products with different fatty acid compositions. This approach enables us to investigate whether the fatty acid composition of nanoemulsions loaded with HON and MAG influences cancer cell response and enhances the effectiveness of chemotherapy in future planned in vivo studies.

#### 2.1.1. Physiochemical Characterization

The applied technological process, based on the low-energy horizontal shaking method, effectively enabled the incorporation of HON, MAG, and their combination (HON-MAG) into both intravenous nanoemulsions (Clinoleic and Lipidem), achieving an encapsulation efficiency (EE%) exceeding 95% for all samples. The physicochemical properties of the resulting formulations are summarized in Table 1.

As shown by the presented data, all obtained formulations exhibited appropriate parameters for intravenous administration, both immediately after preparation and following 30 days of storage at 4 ± 1 °C.

The MDD of such systems is a critical safety parameter in the context of intravenous administration. According to the U.S. Pharmacopeia, the MDD limit determined by the dynamic light scattering (DLS) method is set at 500 nm. All formulations obtained in this study met this acceptance criterion, with MDD values below 241.2 nm for Clinoleic-based formulations and below 209.3 nm for Lipidem-based ones. The MDD of the developed formulations was slightly lower than that of the unmodified commercial products, indicating the lack of the negative effect of added substances on the lipid droplets’ structure and stability. Similarly, the presence of HON and MAG had a negligible effect on the polydispersity index (PDI) and zeta potential. Three-month stability tests showed that the MDD values of nanoemulsions stored under refrigerator conditions (4 ± 1 °C) decreased over time, indicating the dynamics of the applied system and the satisfactory stability of obtained formulations. At the same time, the chemical stability of HON and MAG in the nanoemulsions was verified using the HPLC method. The decrease in the HON and MAG content in the developed formulations during the storage period did not exceed 10% of the initial value.

#### 2.1.2. Biological Activity of HON and MAG in Nanoemulsions

The therapeutic potential of the developed nanoemulsions was evaluated by analyzing their effect on the viability of head and neck cancer cells. Unloaded IV nanoemulsions slightly reduced cell viability across both cancer cell lines. Specifically, for Lipidem, cell viability was 91.83 ± 8.04% in FaDu cells and 83.03 ± 15.3% in SCC-040 cells. For Clinoleic, FaDu cells exhibited 84.42 ± 8.93% viability and SCC-040 cells 83.55 ± 12.08% when compared to untreated control cells.

**Figure 1:**
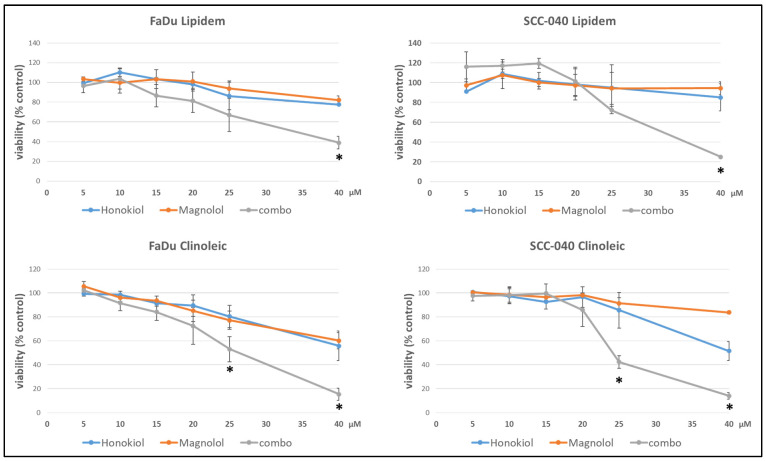
The effect of Lipidem- or Clinoleic-based formulations of HON, MAG, and their combination (combo) on the viability of FaDu or SCC-040 head and neck cancer cells after 48 h incubation. Mean results from four experiments (with nine technical replicates each) are shown ±SD. Statistically significant differences between the effects of both individually applied compounds and their combinations are denoted with an asterisk (*t*-test, *p* ≤ 0.05).

The studied formulations loaded with HON, MAG, and their combination led to comparable effects on cell viability in both cell lines (Figure 1). Lipidem-based formulations containing individual compounds did not exert strong effects within the analyzed concentration range, with viability remaining above 75%. In contrast, Clinoleic-based formulations led to a greater reduction in cell viability at the highest concentrations, though the differences were not statistically significant. The IV nanoemulsions loaded with both phytochemicals more effectively reduced viability than those containing single compounds, with statistically significant differences observed at the highest concentrations. Again, Clinoleic-based formulations encapsulating both compounds appeared more potent than Lipidem-based ones; however, the differences between Lipidem-based and Clinoleic-based nanoemulsions were not statistically significant.

The impact of the developed formulations on cell viability, assessed in FaDu and SCC040 cells, demonstrated a synergistic effect for formulations containing both HON and MAG compared to nanoemulsions with either compound alone. The current study focused on evaluating the cytotoxic effect, disregarding the molecular mechanism underlying the synergistic action of HON and MAG. However, based on the literature data, it can be assumed that this effect may result from several factors. For example, in a study on glioblastoma cells, the combination of HON and MAG was found to reduce the levels of cyclin A, D1, and cyclin-dependent kinases 2, 4, and 6. Additionally, there was a decrease in p-PI3K, p-Akt, and Ki67, resulting in an antiproliferative effect. The combination of MAG and HON induced autophagy and apoptosis [19]. In another study on bladder cancer cells, MAG alone did not produce the expected results. However, when used in combination with HON, there was a decrease in cell viability and cell cycle arrest, as well as caspase-3-dependent apoptosis and autophagy. Using HON and MAG separately did not demonstrate an anti-migratory effect; however, using them in combination inhibited cell motility [20]. Perhaps similar mechanisms also contributed to the observed synergy of MAG and HON in the tested nanoemulsions, but further studies are needed to clarify this.

Notably, Clinoleic-based formulations exhibited greater effects than Lipidem-based formulations. These findings suggest that the composition of fatty acids can directly affect cancer cells, potentially linking this effect to the anticancer properties of oleic acid described in the literature [21,22,23]. Oleic acid has been shown to inhibit cellular proliferation in various tumor cell lines including OE19 and OE33 esophageal cancer cells [22] and tongue squamous cell carcinomas (TSCCs) [23]. The proposed mechanisms of its anticancer activity include suppressing HER2 over-expression, inducing apoptosis through caspase-3 activation and reactive oxygen species generation, and modulating intracellular calcium signaling pathways [21]. Additionally, in human esophageal cancer cells, oleic acid activates tumor suppressor genes (p27, p21, and p53), inhibits cell proliferation, migration, and adhesion, and reduces colony size [22]. In tongue squamous cell carcinoma (TSCC), oleic acid promotes G0/G1 cell cycle arrest, induces autophagy, and enhances apoptosis by inhibiting the Akt/mTOR signaling pathway, as indicated by changes in protein markers such as p-mTOR, p-Akt, and caspase-3 [23]. These anticancer effects, along with the observed decrease in cell viability in both FaDu and SCC-040 cell lines treated with HON- and MAG-containing Clinoleic-based formulations, suggest that the effect may result from the synergistic action of the active compounds and oleic acid present in the nanoemulsion. However, the precise mechanisms underlying this effect require further investigation.

#### 2.1.3. Cell Uptake of HON and MAG from Nanoemulsions

**Figure 2:**
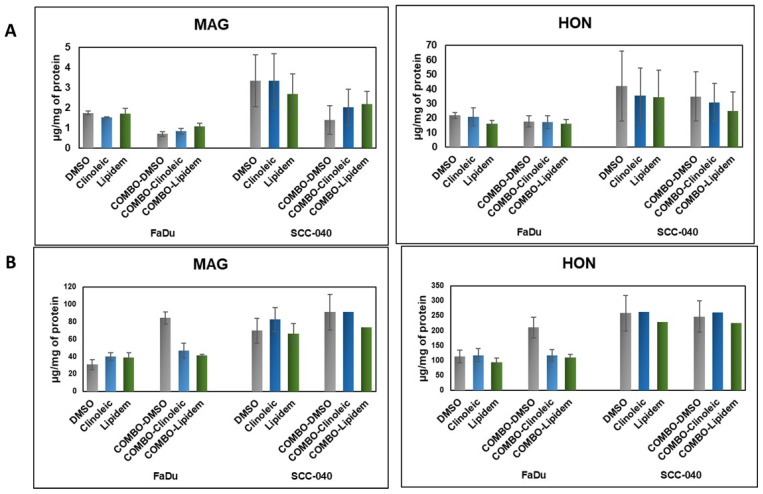
The uptake of HON and MAG from their Lipidem- or Clinoleic-based formulations into FaDu and SCC-040 head and neck cancer cells. (**A**) Concentrations of HON and MAG determined in the cell lysates, reflecting intracellular uptake. (**B**) Concentrations of HON and MAG measured in the culture medium, indicating the remaining extracellular fraction. Data are presented as mean values ± standard deviation (SD) from two independent replicates.

The cellular uptake of HON and MAG following incubation with HON/MAG- or HON-MAG-loaded nanoemulsions was analyzed to confirm the ability of the tested formulations to effectively deliver HON and MAG into the cells. As presented in Figure 2, both compounds were successfully delivered, exhibiting uptake profiles similar to those observed for DMSO solutions.

For both active substances, intracellular concentrations after 2 h of incubation were higher in the SCC-040 cell line than in FaDu. Notably, the HON uptake was nearly 10-fold higher than that of MAG; however, no significant differences were observed in the efficiency of HON and MAG delivery between Clinoleic- and Lipidem-based formulations. When comparing extracellular concentrations of HON and MAG, HON levels were three times higher than those of MAG. This suggests that MAG is more efficiently internalized (as indicated by lower extracellular concentrations) and potentially metabolized more rapidly (reflected by lower intracellular concentrations) than HON. Interestingly, in the FaDu cell line, formulations containing a combination of HON and MAG enhanced the cellular uptake of both compounds, as evidenced by lower concentrations of HON and MAG in the culture medium compared to DMSO-based solutions (Figure 2B).

In summary, despite the substantial differences in uptake levels after 2 h of incubation (Figure 2), both substances exhibited comparable effects on cell viability after 48 h (Figure 1). This indicates that their biological activity may not be directly proportional to their internalization dynamics and resulting intracellular concentrations. Moreover, the lack of significant differences between Clinoleic- and Lipidem-based formulations suggests that the observed effects on cell viability are influenced by mechanisms beyond cellular uptake kinetics. It is likely that the formulation components play a crucial role in modulating these effects, potentially affecting intracellular pathways or enhancing the bioactivity of the compounds.

### 2.2. Evaluation of the Impact of Radiation Sterilization on the Stability of HON and MAG

The preparation of intravenous nanoemulsions loaded with HON, MAG, or their combination involved the horizontal shaking of the active substances in commercial IV nanoemulsions, which are sterile products. This method enables the sterilization of HON and MAG prior to their incorporation into the nanoemulsion system, eliminating the need to sterilize the final product. The European Pharmacopoeia Ph. Eur. 10.0 [24] recommends several methods of drug sterilization, including radiation sterilization. This method utilizes the bactericidal properties of ionizing radiation. Gamma radiation, which employs high-energy photons, and e-beam radiation, which uses a focused stream of electrons, have both been widely validated for their sterilization efficiency, as they can effectively eliminate microbial contaminants without significant elevation in temperature. These methods have demonstrated excellent results in preserving the stability of various compounds while ensuring their sterility, as demonstrated for chloramphenicol, alkaloids, and non-steroidal anti-inflammatory drugs [25,26,27,28].

The deep penetration capability of ionizing radiation ensures uniform sterilization, even in densely packed materials or sealed products. However, despite its effectiveness, ionizing radiation can induce alterations in the chemical structure of irradiated compounds, particularly in the solid state. These changes are influenced by various factors, such as the compound’s chemical structure, the radiation dose, and the type of radiation applied. One major concern is radiodegradation, where exposure to ionizing radiation can cause molecular alterations, including bond cleavage, oxidation, and the formation of free radicals. These modifications may result in the loss of therapeutic efficacy or, in some cases, the generation of undesirable or toxic degradation products.

Therefore, rigorous pharmaceutical analysis is essential to ensure that the sterilized active pharmaceutical ingredients (APIs) maintain their safety, efficacy, and stability [29,30]. The European Pharmacopoeia recommends a standard sterilization dose of 25 kGy to achieve a Sterility Assurance Level (SAL) of 10^−6^ for pharmaceutical products. However, to thoroughly evaluate the potential for structural or chemical changes, higher radiation doses, such as 100 kGy and 400 kGy, are often employed in scientific studies. This approach provides a comprehensive understanding of how ionizing radiation affects the integrity of APIs under extreme conditions.

#### 2.2.1. Visual Examination, PXRD, DSC, and TGA Analysis

HON and MAG, white, odorless, crystalline powders, showed no visible changes in appearance or odor after exposure to ionizing radiation doses ranging from 25 to 400 kGy. Although no macroscopic signs of degradation were observed, advanced analytical methods detected microscopic changes, particularly at higher irradiation doses.

**Figure 3:**
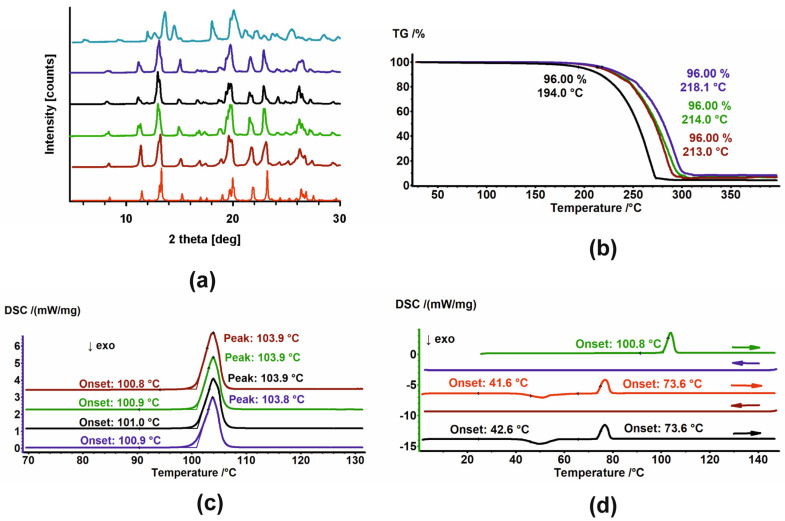
(**a**) PXRD diffractograms; (**b**) TGA and (**c**) DSC thermograms of MAG (brown), MAG 25 kGy (green), MAG 100 kGy (black), and MAG 400 kGy (dark blue). In (**a**), the diffractogram of the sample after heating–cooling cycles in DSC (light blue) and the diffractogram generated based on crystal structures (red) are also shown. (**d**) DSC thermograms of MAG in heating–cooling cycles. The thermograms are arranged sequentially from top to bottom; arrows indicate the direction of temperature change.

**Figure 4:**
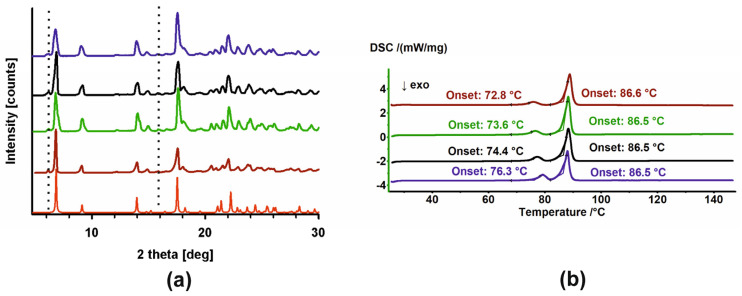
(**a**) PXRD diffractograms and (**b**) DSC thermograms of HON (brown), HON 25 kGy (green), HON 100 kGy (black), and HON 400 kGy (dark blue). In (**a**), the diffractogram generated based on crystal structures (red) is also shown. Additional intensities from the P2 phase are marked with dotted lines.

Samples of HON and MAG were subjected to PXRD and DSC analysis. The thermal stability of MAG powders was additionally assessed by TGA. HON was excluded from TGA experiments because it creates sticky vapors that cause the pan to glue to the thermocouple, which might damage it. The results of PXRD, DSC, and TGA analysis of unirradiated and irradiated MAG are presented in Figure 3, while the PXRD and DSC results for unirradiated and irradiated HON are shown in Figure 4.

**Figure A1:**
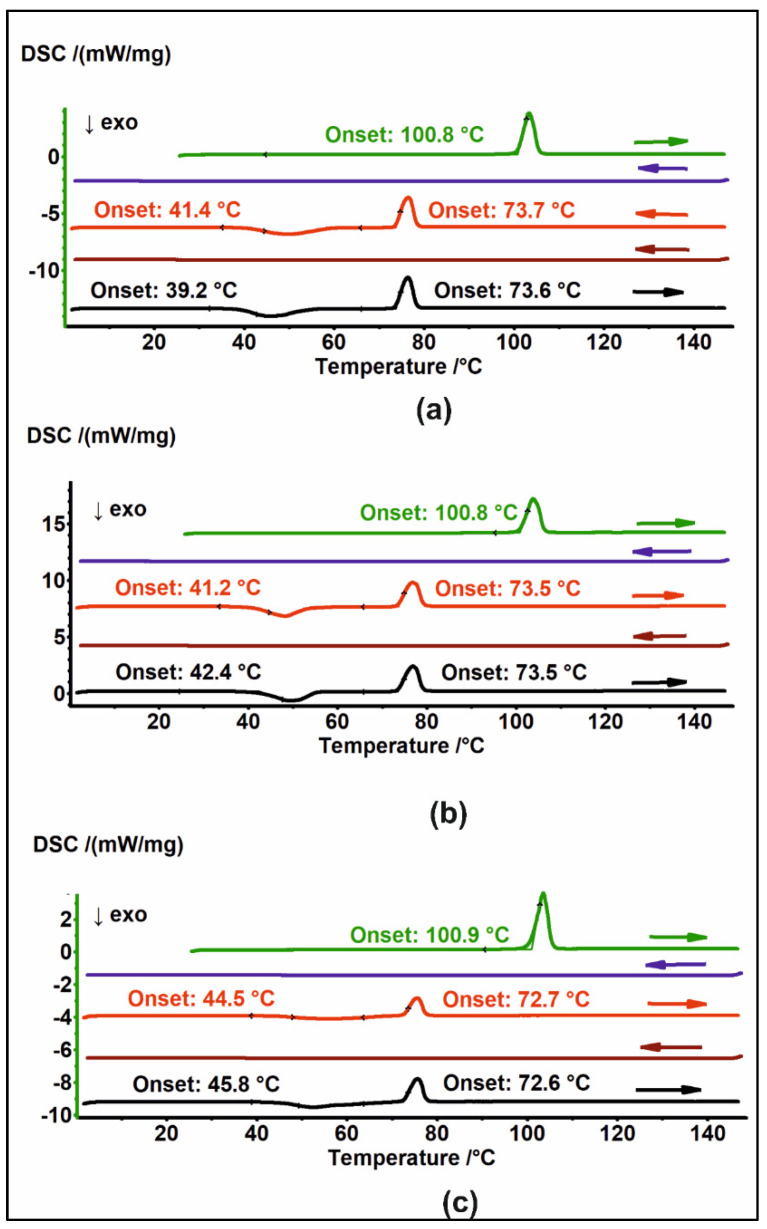
DSC thermograms of (**a**) MAG 25, (**b**) MAG 100, and (**c**) MAG 400 in heating–cooling cycles. The thermograms are arranged sequentially from top to bottom; arrows indicate the direction of temperature change.

The PXRD patterns registered for unirradiated and irradiated samples of MAG showed no changes in the phase composition. Moreover, their comparison with the pattern generated based on crystal structure from the Cambridge Structural Database (CSD) [31,32] indicates the phases’ identity (Figure 3a). Melting points, with an onset around 110.9 °C, assigned using DSC, also confirmed that samples do not change after irradiation (Figure 3c). Additionally, the thermal properties of their melts were the same. No thermic events were detected in the cooling cycles, while during the heating cycles, first, the exothermic peak of cold crystallization of the melt was observed. Second, the melting endotherm with an onset around 73.6 °C was registered (Figure 3d and Figure A1). The significant change in the melting points of the starting materials and the crystalline materials obtained from the melts undoubtedly indicates the formation of a new, metastable polymorph. The PXRD diffractogram registered after the final DSC heating cycle of MAG differed from the starting one, thus corroborating the creation of a new polymorph (Figure 3a, light-blue pattern). Considering these results, the findings from TGA, which show changes in the thermal stability of the samples, are surprising (Figure 3b). MAG irradiated at 100 kGy is significantly less stable compared to the other three powders, though the reason for this remains unclear.

The diffractograms recorded for unirradiated and irradiated HON (Figure 4a) clearly indicated their phase uniformity, so the radiation also did not influence the samples. However, phase analysis demonstrates that these samples comprised two phases (compare the red diffractogram with the others in Figure 4). The major component identified in the CSD database [32] is accompanied by small additional peaks corresponding to a second phase. Further in the text, these phases are denoted as P1 and P2, respectively.

In the DSC thermograms, two endothermic peaks are visible (Figure 4b): first, with the onset that changes from 72.8 °C to 76.3 °C depending on the sample (irradiated or not and the dose of radiation), and second, with the stable onset temperature at 86.5 °C. It appears that irradiation affects only the first process. These diffraction patterns and DSC thermograms have been previously described in the literature [33,34,35]. However, no reports have been found regarding attempts to identify the origins of the additional peaks in the diffractograms or the two events in the DSC. Therefore, we conducted a series of experiments to investigate this.

**Figure 5:**
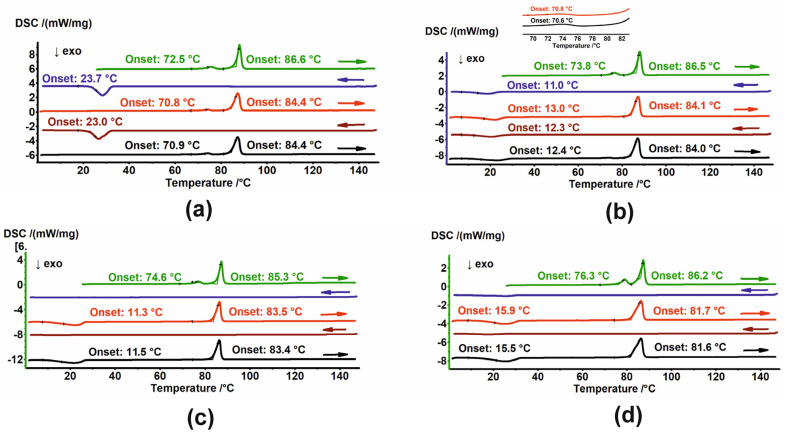
DSC thermograms of (**a**) HON, (**b**) HON 25 kGy, (**c**) HON 100 kGy, and (**d**) HON 400 kGy in heating–cooling cycles. The thermograms are arranged sequentially from top to bottom; arrows indicate the direction of temperature change. In (**b**), a zoomed-in view of the second and third heating cycles is shown at the top.

Similarly to for MAG, the crystallization behavior of molten HON samples, both unirradiated and irradiated, was examined. The DSC thermograms obtained in heating–cooling–heating–cooling–heating cycles are shown in Figure 5. The thermal properties of the melt differ depending on the starting material.

Two endothermic peaks are visible for HON during the second and third heating cycles. Although the temperature shifts to lower values and the first endotherm has a smaller area, i.e., smaller enthalpy, these effects appear to have the same origin as those observed during the first heating. During the cooling cycles, crystallization exotherms with an onset around 23.3 °C are observed (Figure 5a).

While cooling, the crystallization exotherm is also seen for HON 25, but at a lower temperature, with an onset around 11.5 °C. However, during heating, an additional exotherm of cold crystallization, with onset around 12.7 °C, was detected, followed by two endothermic events with the same temperatures as observed for unirradiated HON. Notably, the endotherm with onset just above 70 °C has a lower enthalpy than those registered for unirradiated HON (Figure 5b).

**Figure 6:**
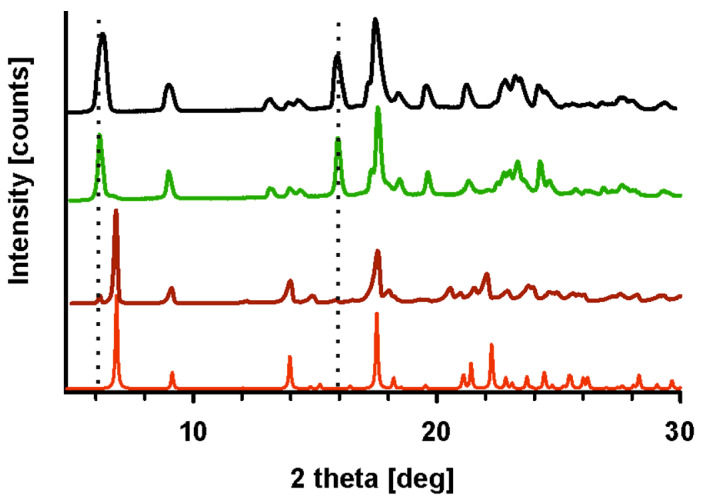
PXRD diffractograms generated based on crystal structures (red), of HON (brown), HON 400 kGy after a few heating–cooling cycles (green), and HON heated to 78 °C (black). Intensities from the P2 phase are marked with dotted lines.

The thermal properties of the HON 100 kGy and 400 kGy melts are similar: no peaks over cooling cycles and one exothermic and one endothermic event during heating (Figure 5c,d). The PXRD diffractogram registered for HON 400 kGy after DSC indicates the change in the proportion of the phases. Now, the second phase, P2, which was in the minority for the starting samples, dominates over phase P1 (Figure 6).

Based on these results, a few conclusions might be drawn. First, the starting phases P1 and P2 are two polymorphic forms of HON, with their proportions varying depending on the conditions. Second, cold crystallization and melting around 81.7 °C characterize phase P2, while crystallization during cooling and the first endothermic peak in the heating cycle are features of P1. Considering that P1 is in the majority in the starting sample, a small enthalpy of the first endotherm suggests that this is not melting but rather a solid-state-to-solid-state phase transformation. To corroborate this conclusion, a PXRD experiment was performed following heating HON to 78 °C, so above the phase transition temperature but below melting. In the resulting diffractogram, only peaks characteristic of P2 are seen; those of P1 disappeared, so the transformation of P1 to P2 is confirmed (Figure 6).

When comparing the effects of the 25 kGy dose to higher irradiation doses, distinct differences emerge. At higher doses, particularly 100 kGy and 400 kGy, thermal changes become more pronounced. For MAG, thermogravimetric analysis showed reduced thermal stability at 100 kGy, an effect not observed at 25 kGy. Additionally, prolonged heating–cooling cycles in DSC led to the formation of a metastable polymorph, as evidenced by changes in PXRD patterns. For HON, higher temperatures caused a shift in the proportions of the two polymorphic phases, P1 and P2. PXRD analysis after heating the unirradiated sample to 78 °C confirmed the complete transformation of P1 to P2 at elevated doses, indicating a greater sensitivity to prolonged thermal stress.

#### 2.2.2. EPR Analysis

**Figure 7:**
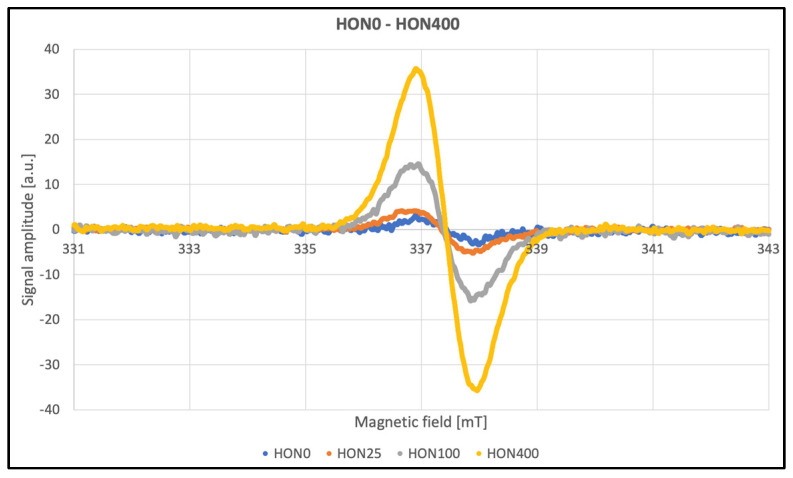

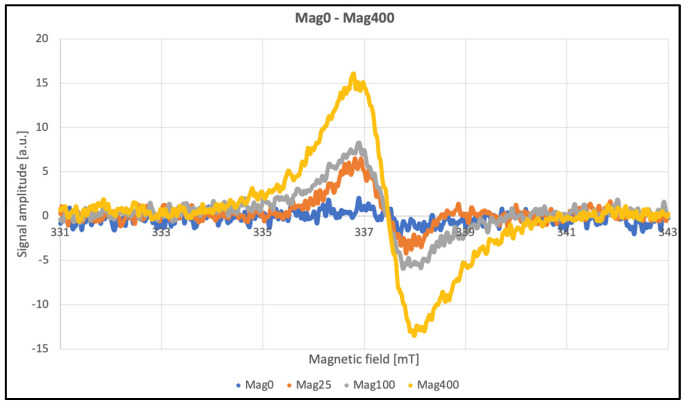
EPR spectra of HON and MAG.

The EPR method was used to detect and characterize free radicals. Overmodulation causes an artificial broadening of the lines, but the signal intensities can still be compared. The EPR spectra for HON and MAG are presented in Figure 7.

The line widths listed in Table 2 contain information about the amplitude of the second modulation, making these parameters unsuitable for meaningful analysis. After approximately three months, repeated tests were conducted to assess the stability of the paramagnetic centers induced by radiation. Data were collected at a modulation amplitude roughly half the line widths to ensure accurate measurements (Table 2). No significant differences were observed as a function of the radiation dose. The average line width for HON samples was approximately 10 G, while for MAG samples, it was 11 G. According to theory, an even eightfold-lower modulation should be used; however, this was not feasible due to signal intensity limitations. Additionally, the obtained line widths were confirmed using the multiharmonic technique. This method allows for the accurate determination of spectral parameters regardless of the second modulation amplitude. Even significant overmodulation does not pose a problem. Another advantage of this technique is the improvement in the signal-to-noise ratio [36].

**Figure 8:**
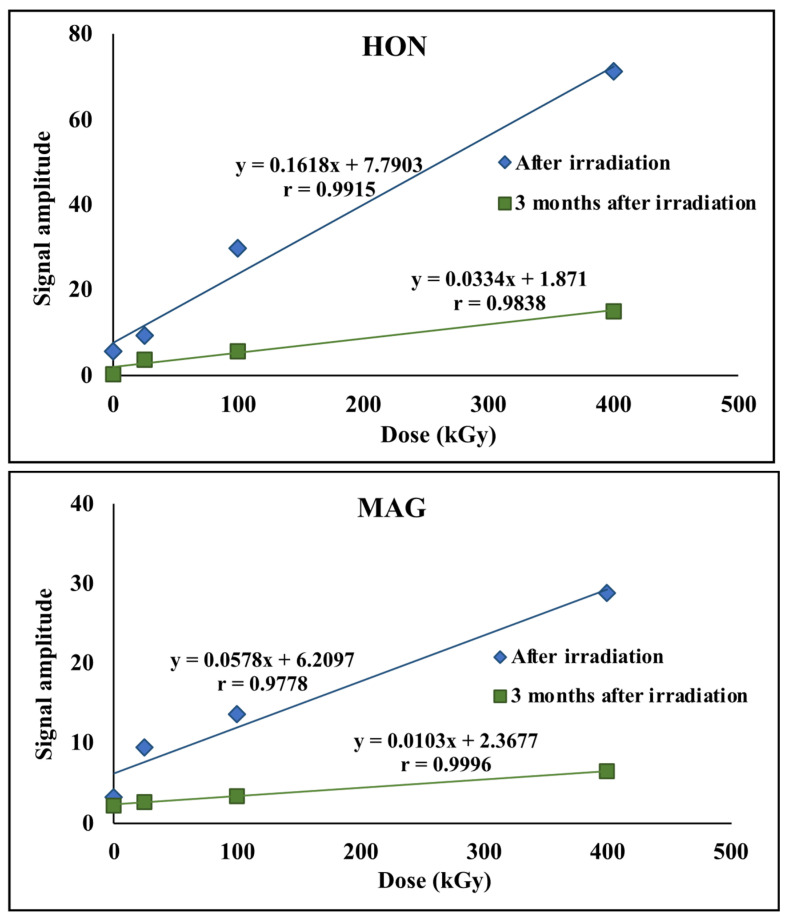
EPR signal amplitudes for HON and MAG.

Significant changes in the amplitude of EPR signals were observed as a function of the ionizing radiation dose applied to the investigated samples. The paramagnetic centers induced by radiation gradually disappeared over time; after approximately three months, the signal amplitudes had decreased by more than 70%, as shown in Figure 8. Other spectral parameters remained unchanged within the limits of measurement error, suggesting that the generated radicals did not cause permanent or significant structural damage.

#### 2.2.3. FT-IR, NMR, and HPLC Analysis

**Figure 9:**
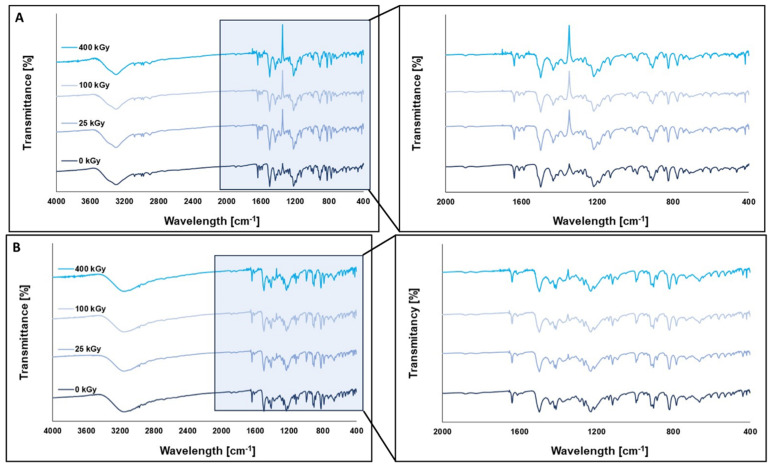
FT-IR spectra of HON (**A**) and MAG (**B**) before and after irradiation at a dose of 25 kGy, 100 kGy, and 400 kGy.

The FT-IR method was used to detect structural or functional group modifications in HON and MAG that may have occurred due to radiation. Additionally, NMR analysis was performed to identify any changes in the molecular structure, chemical environment, or potential formation of degradation products. The results of FT-IR analysis are presented in Figure 9.

The shape and intensity of the FT-IR spectra did not show any significant differences between the unirradiated and irradiated samples, indicating that the FT-IR method is not suitable for detecting structural changes in the tested compounds under the influence of ionizing radiation.

To investigate the impact of ionizing radiation on the molecular dynamics of HON and MAG, spin–lattice relaxation time (T_1_) measurements were performed using ^1^H NMR spectroscopy. The activation parameters describing the molecular dynamics of HON and MAG samples irradiated with 0 kGy and 400 kGy were estimated by analyzing the temperature dependence of the spin–lattice relaxation times T_1_ based on the Bloembergen–Purcell–Pound (BPP) theory [37]. The T_1_ values depend on dipolar interactions modulated by the internal motions of the molecule. These contributions are additive, and the relaxation rate of the multi-proton system can be determined using the formula below [37,38]:

(1) $$\frac{1}{T_{1}}=\frac{2}{3}\gamma^{2}\sum ∆M_{2k}\left(\frac{\tau_{ck}}{1+\omega_{0}^{2}\tau_{ck}^{2}}+\frac{4\tau_{ck}}{1+{4\omega }_{0}^{2}\tau_{ck}^{2}}\right),$$

where γ is the gyromagnetic ratio of protons, ΔM_2k_ is the reduction in the second moment in ^1^H NMR spectra induced by the contributing motion, and τ_ck_ is the correlation time expressed by the Arrhenius equation:

(2) $$\tau_{ck}=\tau_{0}exp\left(\frac{E_{ak}}{RT}\right),$$

where τ_0_ is the pre-exponential factor, E_ak_ is the activation energy of molecular motion, and R is the gas constant.

**Figure 10:**
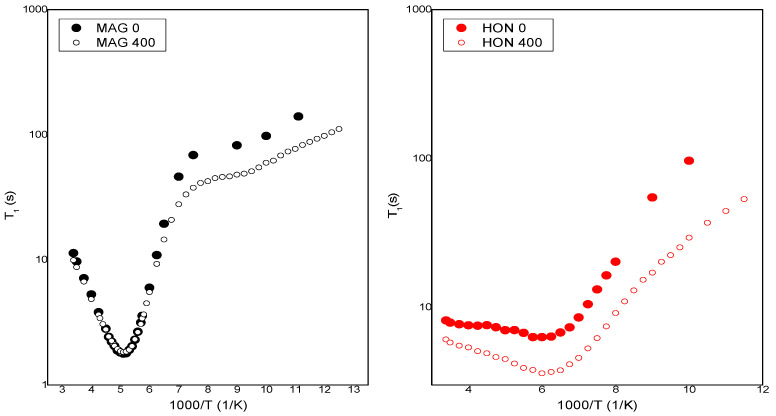
The temperature dependence of spin relaxation time T_1_ for MAG and HON before and after irradiation.

Figure 10 shows the temperature dependence of the spin–lattice relaxation times (T_1_) for MAG and HON before and after irradiation with a dose of 400 kGy. For MAG, the relaxation time values remain unchanged in the temperature range from 295 K to 152 K, with a noticeable shortening of relaxation times for the irradiated sample only at lower temperatures. It is assumed that irradiation can cause the dissociation of intermolecular bonds, leading to an increase in molecular mobility within the system.

**Figure 11:**
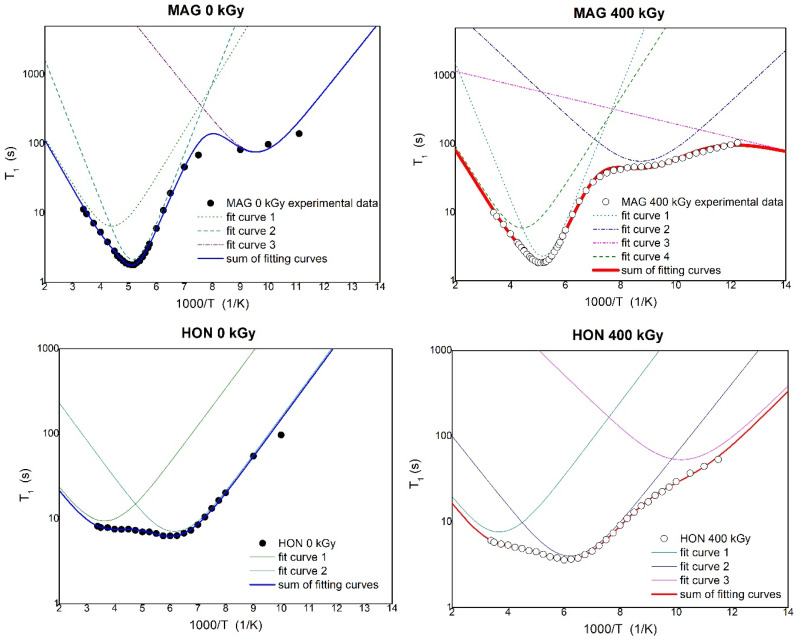
The temperature dependence of spin relaxation time T_1_ for MAG and HON before and after irradiation with fitting of the theoretical relaxation curves to the experimental data.

By fitting Equations (1) and (2) to the experimental data shown in Figure 11, the activation parameters of molecular motions were calculated and are presented in Table 3. In Figure 11, the solid lines represent the best numerical fits to the experimental data based on Equations (1) and (2).

Table 3 presents the activation parameters of the internal motions obtained for MAG and HON before and after irradiation by fitting the theoretical relaxation curve to the experimental data. The uncertainty in the estimated parameters was less than 10%. τ_0_ is the pre-exponential factor, E_ak_ is the activation energy of molecular motions, and ΔM_2k_ is the reduction in the second moment in ^1^H NMR spectra.

The molecular dynamics of HON and MAG, assessed through NMR relaxation studies, revealed increased molecular mobility at higher doses, likely due to the dissociation of intermolecular bonds caused by the greater energy input. This effect was not observed at the 25 kGy dose, where molecular dynamics remained largely unchanged.

Finally, HPLC analysis was performed to quantify the concentration of MAG and HON and assess their chemical stability after exposure to ionizing radiation.

**Figure A2:**
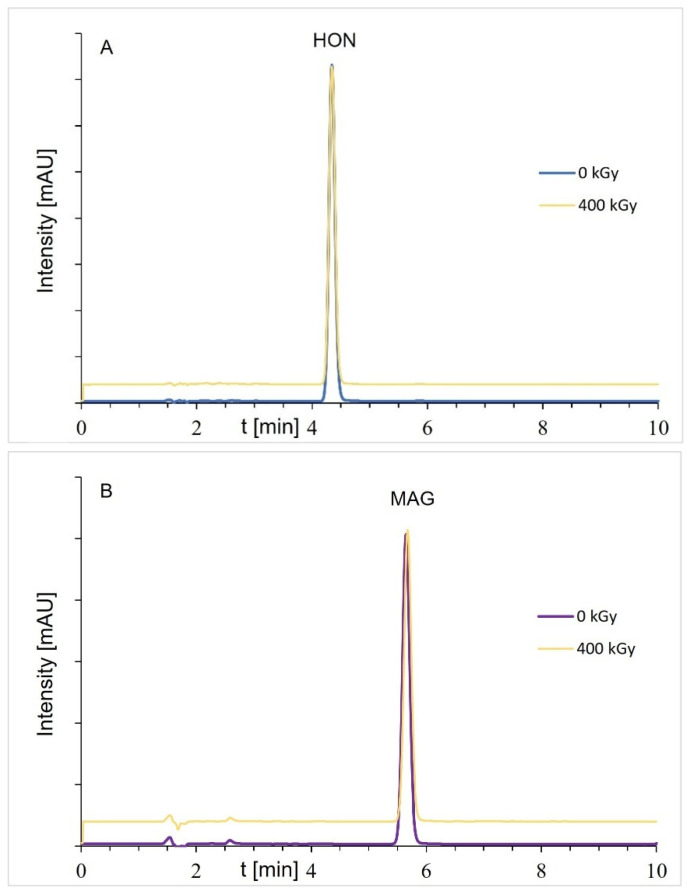
Chromatograms of samples before and after radiation (dose of 400 kGy): (**A**) HON and (**B**) MAG; the HON or MAG concentrations were about 100 μg/mL (DAD detection).

**Figure A3:**
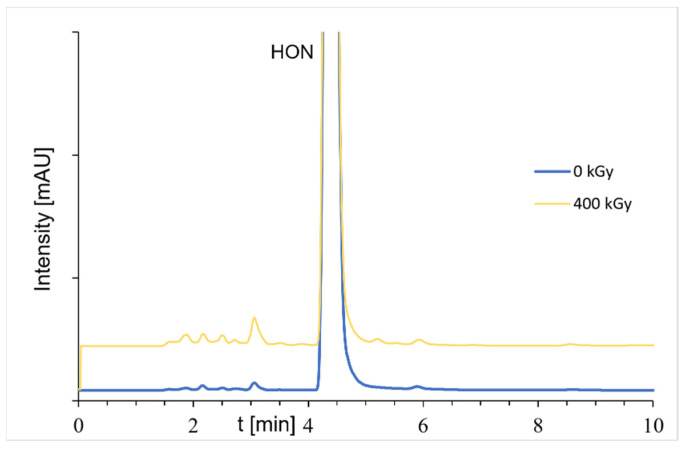
Chromatograms of samples before and after radiation (dose of 400 kGy); the HON concentrations were about 100 μg/mL (FLD detection).

Both HON and MAG maintain high levels of stability across all radiation doses, with HON and MAG content consistently above 95% (Table 4). Moreover, no additional peaks were observed in the chromatograms of the radiation-exposed compounds (examples of chromatograms are presented in Figure A2) by using the DAD detector. In the case of HON, using the FLD detector, the appearance of a few peaks was observed (Figure A3). Some of these peaks are also slightly visible on the chromatogram of the unexposed sample, but the size and number of peaks increase with the greater radiation dose. The sum of the peak areas from all degradation products observed for the irradiated samples is about 1.5 to 5% of the sum of the peaks (unirradiated sample about 0.5%). This observation may indicate an impact of radiation on the chemical stability of HON, especially after the application of a 400 kGy dose.

### 2.3. Limitations and Future Perspective

Despite the promising outcomes, this study has several limitations. The cytotoxicity assays were conducted on only two HNSCC cell lines, which may not fully represent the heterogeneity of the disease. Furthermore, the in vitro nature of the study does not account for the complexities of in vivo systems, including pharmacokinetics, immune responses, and systemic toxicity; therefore, the findings should be considered preliminary and exploratory. Future research should focus on validating these findings in animal models and exploring the long-term stability, immunogenicity, and metabolic fate of the nanoemulsions. The superior performance of Clinoleic-based formulations, compared to Lipidem-based ones, also warrants further investigation to elucidate the role of specific lipid carriers in enhancing therapeutic outcomes. Additionally, the molecular mechanisms underlying the observed synergistic effects of HON and MAG are important directions for further exploration.

## 3. Materials and Methods

### 3.1. Materials

Honokiol (HON) and magnolol (MAG) were sourced from Pol-Aura (Olsztyn, Poland), while Clinoleic and Lipidem were purchased from Baxter (Deerfield, IL, USA) and B. Braun Melsungen AG, (Melsungen, Germany), respectively. All organic solvents used in the study were of analytical or high-performance liquid chromatographic (HPLC) grade. Hypopharyngeal squamous cell carcinoma FaDu cells were procured from the American Type Culture Collection (Manassas, VA, USA), and tongue squamous cell carcinoma SCC-040 cells were obtained from the German Collection of Microorganisms and Cell Cultures (Braunschweig, Germany).

### 3.2. Methods

#### Preparation of IV Nanoemulsions

HON/MAG- and HON-MAG-loaded nanoemulsions were prepared using a previously described low-energy horizontal shaking method [11]. In this process, 26.6 mg (10 mmol) of HON and 26.6 mg (10 mmol) of MAG were accurately weighed into 12 mL glass tubes, which were then filled with 10 mL of Clinoleic or Lipidem and shaken horizontally using a GLF 3005 shaker for 120 min. This preparation process enabled the formulation of IV nanoemulsions containing HON or MAG at concentrations of 10 mM each, as well as a combined formulation with HON and MAG at 10 mM + 10 mM. Following the incorporation procedure, the samples were stored at 4 ± 1 °C for 24 h to allow unincorporated substances to settle. The emulsions were then filtered through a 0.45 µm cellulose filter to remove any residues. The emulsions were stored in the refrigerator at 4 ± 1 °C for 3 months to assess initial stability.

### 3.3. Characterization of Developed IV Nanoemulsions

#### 3.3.1. Mean Droplet Diameter (MDD), Polydispersity Index (PDI), and Zeta Potential (ZP) Analysis

The mean droplet diameter (MDD), polydispersity index (PDI), and zeta potential (ZP) of the HON/MAG- and HON-MAG-loaded nanoemulsions were measured using a Malvern Zetasizer Nano ZS (Malvern Instruments, Malvern, UK). Dynamic light scattering (DLS) and electrophoretic light scattering (ELS) techniques were employed for the determination of MDD and ZP, respectively. Each formulation (100 µL) was diluted with deionized water to a final volume of 10 mL and transferred into specialized polycarbonate cuvettes for MDD, PDI, and ZP analysis. All measurements were performed in triplicate.

#### 3.3.2. Entrapment Efficiency (EE%)

The EE% of HON and MAG in the nanoemulsions was determined using an HPLC method. Chromatographic analysis was conducted on an Agilent 1260 Infinity II LC System (Agilent Technologies, Bolinen, Germany), equipped with a quaternary pump (model G7111B) and degasser, a vial sampler (model G7129A) maintained at 15 ± 2 °C, a multicolumn thermostat (model G7116A) set at 30 ± 0.8 °C, and a diode array detector (model G7115A). The detection wavelength was set to 290 nm. Separation was achieved on a reverse-phase column (C-18(2) 100 Å Luna, 150 × 4.6 mm ID, 5 μm, Phenomenex, Torrance, CA, USA) using an isocratic solvent system consisting of 0.4% acetic acid (21%), acetonitrile (25%), and methanol (54%) as the mobile phase. The injection volume was 10 μL, and the total analysis time was 8 min. The percentage of EE% was determined using the following equation [11]:

$$EE\%=\frac{Amount\;of\;HON\;or\;MAG\;entrapped}{Total\;amount\;of\;HON\;or\;MAG\;added\;to\;PN\;emulsion}\times 100\%$$

#### 3.3.3. The Effect of Developed IV Nanoemulsions on Cell Viability

Hypopharyngeal squamous cell carcinoma FaDu cells and tongue squamous cell carcinoma SCC-040 cells were cultured under standard conditions (37 °C, 5% CO_2_, 90% humidity) using high-glucose DMEM (Capricorn Scientific, Ebsdorfergrund, Germany) supplemented with 5% fetal bovine serum (EURx, Gdańsk, Poland) and 1% antibiotic (penicillin/streptomycin) solution (Biowest, Nuaillé, France). The resazurin assay was used to assess the effect of HON or MAG or HON&MAG-loaded nanoemulsions on cell viability according to the standard protocol. Briefly, the cells were seeded in black 96-well microplates (6 × 10^3^ cells per well). After overnight incubation, fresh medium containing the studied emulsion formulations loaded with HON and MAG at concentrations ranging from 5 to 40 µM was added into wells. Control wells were treated with medium containing IV nanoemulsions without HON and MAG. Additionally untreated control wells were included to assess the effect of the studied nanoemulsions on cell viability. After 48 h, cell viability was evaluated using the resazurin assay. For this purpose, wells were rinsed with PBS buffer, and fresh medium containing the resazurin salt (Sigma-Aldrich, St. Louis, MO, USA) was added. After a two-hour incubation, the fluorescence levels of resorufin were measured (ex—530 nm, em—590 nm) using the Infinite M200 microplate reader (Tecan, Grödig, Austria). The experiment was repeated four times, with nine replicates per experiment.

#### 3.3.4. Cellular Uptake of HON and MAG from Developed IV Nanoemulsions

FaDu and SCC-040 cells were seeded into a six-well plate at a density of 2.5 × 10^5^ cells per well and allowed to grow for 24 h. Subsequently, cells were treated with free substances (HON and MAG dissolved in 1 mL of DMSO and then diluted with the growth medium) or with HON/MAG- or HON-MAG-loaded nanoemulsions at a concentration of 50 µM of HON and MAG and incubated at 37 °C for 2 h. Control cells were treated with the complete growth medium containing 0.2% of DMSO. After incubation, cells were scraped, washed twice with ice-cold PBS, and collected by centrifugation (4 °C, 4000× *g*). The cell pellets were resuspended in 500 µL HPLC mobile phase (acetonitrile and 0.4% acetic acid (60:40 *v*/*v*)) and disrupted by an ultrasonic cell disruptor (Bioruptor Next Generation UCD300, Diagenode, Liège, Belgium) for 1 min. Then, samples were centrifuged for 10 min (4 °C, 15,000× *g*), and 400 µL of the supernatant was filtered and analyzed by HPLC-FLD. Intracellular HON and MAG contents were normalized to the cellular protein content, which was measured using the BCA method (Pierce BCA Protein Assay Kit, ThermoFisher, Rockford, IL, USA).

### 3.4. Development of HON and MAG Sterilization Method

Approximately 0.5 g each of HON and MAG was placed in colorless 5 mL glass vials sealed with plastic stoppers. The samples were irradiated using a linear electron accelerator (LAE 13/9) with a 9.96 MeV electron beam and a current intensity of 6.2 mA until they absorbed doses of 25, 100, and 400 kGy.

### 3.5. Assessment of Irradiated HON and MAG

#### 3.5.1. Powder Diffraction Experiments (PXRD)

Powder X-ray diffraction data were collected using a Bruker AXS D2 Phaser diffractometer (Bruker, Karlsruhe, Germany) with Cu Kα radiation. Before the X-ray diffraction experiments, unirradiated and irradiated HON and MAG were ground with a mortar and pestle. Measurements were performed using sample holders with a 20 mm diameter and 1 mm depth or, for smaller samples, a holder without a hole. Scans were collected with a 2θ range between 5 and 35°, a step size of 0.02°, a counting rate of 2 s/step, and operating conditions of 30 kV and 10 mA. Samples were spun at 30 rpm. A 1 mm slit module was used during measurements.

#### 3.5.2. Differential Scanning Calorimetry (DSC)

Differential scanning calorimetry thermograms were recorded using DSC 214 Polyma (NETZSCH, Selb, Germany). Unirradiated and irradiated HON and MAG samples were sealed in aluminum pans with pierced lids. The nitrogen atmosphere (30 mL/min) was maintained during measurements. The scans were made with a heating rate of 5 K/min. Additionally, DSC measurements in heating–cooling–heating–cooling–heating cycles were performed to check whether the behavior of the melt of all samples was the same. During these measurements, the three-minute isotherms were applied after each dynamic segment.

#### 3.5.3. Thermogravimetry (TGA)

Thermogravimetric analysis was performed using a TG 209 F3 Tarsus instrument (NETZSCH, Selb, Germany). An open corundum crucible was used for the measurements. Unirradiated and irradiated HON and MAG were heated at a rate of 5 K/min under a nitrogen atmosphere (30 mL/min), and their thermograms were collected.

#### 3.5.4. Electron Paramagnetic Resonance (EPR) Spectroscopy

EPR measurements were performed using an Adani X-band spectrometer using eSpinoza control software v1.0.35.2. The spectrometer’s operating parameters are listed in Table 5. Unirradiated and irradiated HON and MAG samples were weighed and placed in thin-walled glass tubes with a 5 mm diameter. A standard tetracyanoquinodimethane sample with a very narrow spectral line was used and secured against the possibility of changing the position. Such a procedure allowed comparison of individual spectra from the side of the cavity, and it was not necessary to consider the resonance cavity’s quality factor in the analysis. During the analysis, individual spectra were scaled to obtain the same amplitudes of the reference spectrum. The reference spectrum was then subtracted.

#### 3.5.5. Fourier-Transform Infrared (FT-IR) Spectroscopy

FT-IR spectra were collected on an IRAffinity-IS Fourier Transform Infrared Spectrophotometer (Shimadzu, Kyoto, Japan) instrument in the range of 4000–400 cm^−1^, with a resolution of 4.0 cm^−1^ and 40 scans. For tablet preparation, 1 mg of unirradiated or irradiated HON and MAG was weighed and combined with 300 mg of KBr, and then IR spectra were recorded.

#### 3.5.6. Nuclear Magnetic Resonance (NMR)

Solid-state ^1^H NMR measurements of spin–lattice relaxation times T_1_ in the laboratory frame were carried out on a pulse spectrometer operating at 25 MHz (El-Lab Tel-Atomic, Poznań, Poland) [38]. The samples with ionizing radiation of 0 kGy and 400 kGy were sealed in glass tubes after being degassed to avoid humidity effects and remove paramagnetic oxygen. Spin–lattice relaxation times T_1_ were measured using the saturation recovery sequence within a temperature range of 80 K to 300 K. T_1_ values were determined by fitting a single-exponential equation, M_0_(1 − exp(−t/T_1_)), to the recovery of the magnetization (M_z_) as a function of time (t), where M_0_ represents the equilibrium magnetization. All measurements were performed with a ± 10% uncertainty.

#### 3.5.7. High-Performance Liquid Chromatography (HPLC)

The HPLC method described for the evaluation of entrapment efficiency (Section 3.3.2.) was used to determine the HON and MAG content before and after radiation exposure (doses: 25; 100; 400 kGy). Minor changes were introduced in the methodology related to the type of detection and the vial sampler temperature (25 °C ± 0.8 °C). Quantitative determinations of MAG and HON were carried out at detection wavelengths 240 nm and 255 nm, respectively. Additionally, an FLD detector was used for qualitative analysis (detection of potential degradation products). The HPLC methods were validated according to the International Council for Harmonisation Requirements (ICH Q2R2) [39]. Stock solutions (~0.2 mg/mL) were prepared by dissolving HON or MAG in methanol and diluted within calibration ranges (n = 9 for HON, n = 7 for MAG) during validation. Precision and accuracy samples (~100 μg/mL) were prepared in nine replicates. Test samples were obtained by dissolving 10 mg of MAG or HON post-radiation in 10 mL of methanol (n = 3), and then diluting to ~100 μg/mL (three replicates were performed for each sample; n = 9). Reference solutions (~100 μg/mL) were prepared and analyzed alongside test samples. Results are expressed as mean content ± SD [%] of the studied substances, calculated from standard solutions (~100 μg/mL). Two standard solutions were prepared and analyzed simultaneously with tested samples.

The methods were linear in the ranges of 5–160 μg/mL for HON (y = 24.937 ± 0.311x; r = 0.9999), and 7.5–149 μg/mL for MAG (y = 22.202 ± 0.375x; r = 0.9999). The limits of detection were 1.5 μg/mL and 1.8 μg/mL, and the limits of quantitation were 4.5 μg/mL and 5.5 μg/mL for HON and MAG, respectively. The intra- and inter-day precision and accuracy studies were carried out for the tested compounds at 100 μg/mL, each with nine samples, in two series. The precision of the methods, expressed by values of relative standard deviation in percentages, was satisfactory (Table A1). Moreover, the methods were accurate, as evidenced by the recovery levels of 99.92–103.13%.

## 4. Conclusions

In summary, this study provides compelling evidence supporting the therapeutic potential of HON and MAG in the treatment of HNSCC. The findings highlight that nanoemulsion-based formulations prepared using Clinoleic and Lipidem exhibit promising physicochemical properties and therapeutic efficacy. Notably, the combination of HON and MAG demonstrated superior cytotoxic effects on HNSCC cell lines compared to individual compounds, underscoring a potential synergistic interaction. Furthermore, Clinoleic-based formulations were superior to Lipidem-based ones, suggesting a role of omega-9 fatty acids in the anticancer effect of the prepared nanoemulsions. Last but not least, the irradiation dose of 25 kGy effectively sterilized HON and MAG while preserving their structural integrity, thermal properties, and stability, with only transient free radical generation. This rigorous analytical assessment was crucial in validating the use of irradiation as a viable sterilization method for HON and MAG and their further incorporation into commercial IV nanoemulsions for in vivo testing.

## Figures and Tables

**Table 1:** Key physicochemical parameters for developed IV nanoemulsions.

Sample	MDD ± SD [nm]	PDI ± SD	ZP ± SD [mV]	EE%± SD [%]
**Results upon preparation**				
Clinoleic	243.1 ± 0.7	0.097 ± 0.018	−27.9 ± 0.8	-
Lipidem	210.7 ± 5.1	0.075 ± 0.009	−28.9 ± 0.5	-
HON–Clinoleic	239.3 ± 4.6	0.103 ± 0.008	−26.1 ± 0.7	97.45 ± 3.98
HON–Lipidem	208.4 ± 3.6	0.102 ± 0.008	−27.7 ± 0.6	101.17 ± 0.75
MAG–Clinoleic	238.4 ± 2.5	0.121 ± 0.006	−29.0 ± 0.6	100.31 ± 0.34
MAG–Lipidem	209.3 ± 2.0	0.101 ± 0.006	−31.3 ± 2.9	101.11 ± 0.21
HON-MAG–Clinoleic	241.2 ± 2.3	0.137 ± 0.011	−30.7 ± 0.6	97.02 ± 3.59 (HON) 100.59 ± 3.70 (MAG)
HON-MAG–Lipidem	209.0 ± 3.2	0.082 ± 0.016	−31.3 ± 1.4	96.75 ± 1.97 (HON) 99.95 ± 1.17 (MAG)
**Results after 3 months of storage**				
Clinoleic	224.1 ± 2.8	0.090 ± 0.005	−23.6 ± 0.5	-
Lipidem	196.9 ± 2.4	0.064 ± 0.018	−25.9 ± 0.6	-
HON–Clinoleic	236.6 ± 1.2	0.107 ± 0.015	−27.1 ± 0.7	97.18 ± 5.09
HON–Lipidem	203.5 ± 2.0	0.098 ± 0.018	−27.8 ± 0.5	99.26 ± 0.25
MAG–Clinoleic	231.2 ± 1.3	0.089 ± 0.005	−27.0 ± 0.5	99.24 ± 2.55
MAG–Lipidem	203.0 ± 0.8	0.095 ± 0.007	−28.9 ± 1.8	100.07 ± 1.03
HON-MAG–Clinoleic	231.0 ± 4.6	0.121 ± 0.024	−29.1 ± 0.7	97.87 ± 2.39 (HON) 99.57 ± 5.49 (MAG)
HON-MAG–Lipidem	203.1 ± 1.5	0.090 ± 0.005	−30.0 ± 0.3	95.79 ± 0.25 (HON) 99.79 ± 0.46 (MAG)

**Table 2:** Results of EPR analysis.

Parameter	Irradiation Dose [kGy]							
	0	25	100	400	0	25	100	400
	HON				MAG			
g-factor	2.0031	2.0032	2.0030	2.0030	2.0028	2.0034	2.0030	2.0026
dH [mT]	1.01	1.08	1.07	1.04	1.10	1.12	1.10	1.25
Signal amplitude	5.7	9.4	29.8	71.2	3.2	9.5	13.7	28.8
Signal amplitude after 3 months	0.4	3.8	5.8	15.0	2.3	2.7	3.4	6.5
Line center [mT]	337.43	337.34	337.41	337.43	337.53	337.31	337.51	337.49

**Table 3:** Activation parameters of the internal motions obtained for MAG and HON before and after irradiation.

Sample	Motion	τ_0_ [s]	E_ak_ [kJ/mol]	ΔM_2k_ [Gs^2^]
MAG 0 kGy	1st	4.45 × 10^−12^	12.77	0.036
	2nd	2.12 × 10^−14^	19.55	0.110
	3rd	5.83 × 10^−14^	9.66	0.003
MAG 400 kGy	1st	6.25 × 10^−12^	12.09	0.039
	2nd	2.72 × 10^−14^	19.09	0.101
	3rd	2.07 × 10^−12^	7.19	0.004
	4th	1.41 × 10^−12^	1.85	0.164
HON 0 kGy	1st	9.99 × 10^−11^	8.40	0.024
	2nd	7.24 × 10^−12^	8.49	0.033
HON 400 kGy	1st	9.99 × 10^−11^	8.28	0.030
	2nd	1.10 × 10^−11^	7.85	0.058
	3rd	2.31 × 10^−12^	6.07	0.004

**Table 4:** Determination of HON and MAG in samples after radiation.

	Average Amount ± SD [%]	
Dose of Radiation	HON	MAG
0 kGy	101.52 ± 2.32 *	100.68 ± 1.54 *
25 kGy	95.99 ± 0.96 **	101.37 ± 0.97 **
100 kGy	97.65 ± 1.44 **	98.40 ± 1.22 **
400 kGy	95.78 ± 0.95 **	95.13 ± 0.97 **

The results are presented as a mean value ± standard deviation (SD) from nine measurements * expressed as a percentage of theoretical content; ** relative to the unirradiated substance.

**Table 5:** The EPR spectrometer’s working parameters.

Parameter	Value
Modulation frequency [kHz]	100,000
Center field [mT]	338
Sweep width [mT]	15
Sweep time [s]	60
Time constant [s]	0.008
Second modulation amplitude [G]	8
Radio frequency power [mW]	0.291
Radio frequency [GHz]	9.460048
Temperature [K]	296.15

## Data Availability

The datasets generated and/or analyzed during the current study are available in the OSF repository, at the following link: https://osf.io/c95bv/?view_only=64416609bb4340c5aa6e940f34d5ada1 (accessed on 31 July 2025).

## Appendix A

**Table A1:** The precision and accuracy of the HPLC methods of HON and MAG determination after radiation.

Precision/Recovery					
	Intra-Day				Inter-Day
	Series I n = 9		Series II n = 9		Series I–II n = 18
	Concentration [μg/mL]	RSD [%] Recovery [%]	Concentration [μg/mL]	RSD [%] Recovery [%]	RSD [%] Recovery [%]
**HON**	100.3	1.57 103.13 ± 1.24	100.1	1.73 99.92 ± 1.33	2.28 101.52 ± 1.15
**MAG**	100.0	1.52 101.23 ± 1.28	100.3	1.42 100.13 ± 1.19	1.53 100.68 ± 0.82

Results of recovery are expressed as a percentage average amount of API ± value of absolute error. RSD [%]—relative standard deviation in percentages.

## References

[1] Sarrica A., Kirika N., Romeo M., Salmona M., Diomede L. (2018). Safety and Toxicology of Magnolol and Honokiol. Planta Med..

[2] Zhang J., Chen Z., Huang X., Shi W., Zhang R., Chen M., Huang H., Wu L. (2019). Insights on the Multifunctional Activities of Magnolol. BioMed Res Int..

[3] Rauf A., Olatunde A., Imran M., Alhumaydhi F.A., Aljohani A.S.M., Khan S.A., Uddin M.S., Mitra S., Emran T.B., Khayrullin M. (2021). Honokiol: A Review of Its Pharmacological Potential and Therapeutic Insights. Phytomedicine.

[4] Dominiak K., Gostyńska A., Szulc M., Stawny M. (2024). The Anticancer Application of Delivery Systems for Honokiol and Magnolol. Cancers.

[5] Elhabak M., Osman R., Mohamed M., El-Borady O.M., Awad G.A.S., Mortada N. (2020). Near IR responsive targeted integrated lipid polymer nanoconstruct for enhanced magnolol cytotoxicity in breast cancer. Sci. Rep..

[6] Wang Y., Sun C., Huang L., Liu M., Li L., Wang X., Wang L., Sun S., Xu H., Ma G. (2022). Magnolol-loaded cholesteryl biguanide conjugate hydrochloride nanoparticles for triple-negative breast cancer therapy. Int. J. Pharm..

[7] Kleszcz R., Dorna D., Stawny M., Paluszczak J. (2024). Honokiol Is More Potent than Magnolol in Reducing Head and Neck Cancer Cell Growth. Curr. Issues Mol. Biol..

[8] Preeti, Sambhakar S., Malik R., Bhatia S., Al Harrasi A., Rani C., Saharan R., Kumar S., Geeta, Sehrawat R. (2023). Nanoemulsion: An Emerging Novel Technology for Improving the Bioavailability of Drugs. Scientifica.

[9] Sampieri-Morán J.M., Bravo-Alfaro D.A., Uribe-Lam E., Luna-Barcenas G., Montiel-Sánchez M., Velasco-Rodríguez L.D.C., Acosta-Osorio A.A., Ferrer M., García H.S. (2025). Delivery of Magnolia Bark Extract in Nanoemulsions Formed by High and Low Energy Methods Improves the Bioavailability of Honokiol and Magnolol. Eur. J. Pharm. Biopharm..

[10] Bharti B., Li H., Ren Z., Zhu R., Zhu Z. (2022). Recent advances in sterilization and disinfection technology: A review. Chemosphere.

[11] Gostyńska A., Czerniel J., Kuźmińska J., Żółnowska I., Brzozowski J., Krajka-Kuźniak V., Stawny M. (2023). The Development of Magnolol-Loaded Intravenous Emulsion with Low Hepatotoxic Potential. Pharmaceuticals.

[12] Candiloro F., Borioli V., Borsellino G., Picozza M., Pellini R., Cereda E., Gargano F., Caraccia M., Nardi M.T., Bellu L. (2021). Influence of different lipid emulsions on specific immune cell functions in head and neck cancer patients receiving supplemental parenteral nutrition: An exploratory analysis. Nutrition.

[13] Serhan C.N. (2014). Pro-resolving lipid mediators are leads for resolution physiology. Nature.

[14] Yan D., Ye S., He Y., Wang S., Xiao Y., Xiang X., Deng M., Luo W., Chen X., Wang X. (2023). Fatty acids and lipid mediators in inflammatory bowel disease: From mechanism to treatment. Front. Immunol..

[15] Canhada S., Castro K., Perry I.S., Luft V.C. (2018). Omega-3 fatty acids’ supplementation in Alzheimer’s disease: A systematic review. Nutr. Neurosci..

[16] Freitas R.D.S., Campos M.M. (2019). Protective Effects of Omega-3 Fatty Acids in Cancer-Related Complications. Nutrients.

[17] Matsui R., Sagawa M., Sano A., Sakai M., Hiraoka S.I., Tabei I., Imai T., Matsumoto H., Onogawa S., Sonoi N. (2024). Impact of Perioperative Immunonutrition on Postoperative Outcomes for Patients Undergoing Head and Neck or Gastrointestinal Cancer Surgeries: A Systematic Review and Meta-analysis of Randomized Controlled Trials. Ann. Surg..

[18] Aslan C., Maralbashi S., Shekari N., Javadian M., Shomali N., Kazemi T. (2024). Differential effects of docosahexaenoic acid (DHA) and linoleic acid (LA) on miR-101 and miR-342 tumor suppressor microRNAs in Taxol-treated HER2-positive breast cancer cells. Clin. Nutr. ESPEN.

[19] Cheng Y.C., Hueng D.Y., Huang H.Y., Chen J.Y., Chen Y. (2016). Magnolol and honokiol exert a synergistic anti-tumor effect through autophagy and apoptosis in human glioblastomas. Oncotarget.

[20] Wang H.-H., Chen Y., Changchien C.-Y., Chang H.-H., Lu P.-J., Mariadas H., Cheng Y.-C., Wu S.-T. (2020). Pharmaceutical Evaluation of Honokiol and Magnolol on Apoptosis and Migration Inhibition in Human Bladder Cancer Cells. Front. Pharmacol..

[21] Carrillo C., Cavia M.D.M., Alonso-Torre S.R. (2012). Antitumor effect of oleic acid; mechanisms of action: A review. Nutr. Hosp..

[22] Moon H.S., Batirel S., Mantzoros C.S. (2014). Alpha linolenic acid and oleic acid additively down-regulate malignant potential and positively cross-regulate AMPK/S6 axis in OE19 and OE33 esophageal cancer cells. Metabolism.

[23] Jiang L., Wang W., He Q., Wu Y., Lu Z., Sun J., Liu Z., Shao Y., Wang A. (2017). Oleic acid induces apoptosis and autophagy in the treatment of Tongue Squamous cell carcinomas. Sci. Rep..

[24] Deutscher Apotheker Verlag (2019). European Pharmacopea.

[25] Marciniec B., Stawny M., Kozak M., Naskrent M. (2006). Theeffect of ionizing radiation on chloramphenicol. J. Therm. Anal. Calorim..

[26] Marciniec B., Kozak M., Naskrent M., Hofman M., Dettlaff K., Stawny M. (2009). DSC and EPR analysis of some radiation sterilized alkaloids. J. Therm. Anal. Calorim..

[27] Katušin-Ražem B., Hamitouche K., Maltar-Strmečki N., Kos K., Pucić I., Britvić-Budicin S., Ražem D. (2005). Radiation sterilization of ketoprofen. Radiat. Phys. Chem..

[28] Janiaczyk M., Jelińska A., Woźniak-Braszak A., Bilski P., Popielarz-Brzezińska M., Wachowiak M., Baranowski M., Tomczak S., Ogrodowczyk M. (2022). Electron Beam Radiation as a Safe Method for the Sterilization of Aceclofenac and Diclofenac-The Usefulness of EPR and 1H-NMR Methods in Determination of Molecular Structure and Dynamics. Pharmaceutics.

[29] Bakhrushina E.O., Afonina A.M., Mikhel I.B., Demina N.B., Plakhotnaya O.N., Belyatskaya A.V., Krasnyuk I.I., Krasnyuk I.I. (2024). Role of Sterilization on In Situ Gel-Forming Polymer Stability. Polymers.

[30] Van Cauwenbergh T., Theys E., Stroeykens D., Croonenborghs B., Gillet A., DeMent A., Van Schepdael A., Haghedooren E. (2022). The Effect of Gamma and Ethylene Oxide Sterilization on a Selection of Active Pharmaceutical Ingredients for Ophthalmics. J. Pharm. Sci..

[31] Wang Y., Cheng M., Lee J., Chen F. (1983). Molecular and Crystal Structure of Magnolol—C_18_ H_18_ O_2_. J. Chin. Chem. Soc..

[32] Groom C.R., Bruno I.J., Lightfoot M.P., Ward S.C. (2016). The Cambridge Structural Database. Acta Crystallogr. B Struct. Sci. Cryst. Eng. Mater..

[33] Lu X., Lu X., Zhang Z., Lv H. (2020). Preparation and Characterization of Honokiol Nanosuspensions and Preliminary Evaluation of Anti-Inflammatory Effect. AAPS PharmSciTech.

[34] Han M., Yu X., Guo Y., Wang Y., Kuang H., Wang X. (2014). Honokiol nanosuspensions: Preparation, increased oral bioavailability and dramatically enhanced biodistribution in the cardio-cerebro-vascular system. Colloids Surf. B Biointerfaces.

[35] He Y., Hou X., Guo J., He Z., Guo T., Liu Y., Zhang Y., Zhang J., Feng N. (2020). Activation of a gamma–cyclodextrin–based metal–organic framework using supercritical carbon dioxide for high–efficient delivery of honokiol. Carbohydr. Polym..

[36] Gonet M., Baranowski M., Czechowski T., Kucinska M., Plewinski A., Szczepanik P., Jurga S., Murias M. (2020). Multiharmonic electron paramagnetic resonance imaging as an innovative approach for in vivo studies. Free Radic. Biol. Med..

[37] Bloembergen N., Purcell E.M., Pound R.V. (1948). Relaxation Effects in Nuclear Magnetic Resonance Absorption. Phys. Rev..

[38] Kubo R., Tomita K. (1954). A General Theory of Magnetic Resonance Absorption. J. Phys. Soc. Jpn..

[39] ICH Q2(R2) Validation of Analytical Procedures—Scientific Guideline European Medicines Agency. https://www.ema.europa.eu/en/ich-q2r2-validation-analytical-procedures-scientific-guideline.

